# Vitamin D deficiency in schizophrenia implications for COVID-19 infection

**DOI:** 10.1017/ipm.2020.107

**Published:** 2020-09-11

**Authors:** D. Viani-Walsh, S. Kennedy-Williams, D. Taylor, F. Gaughran, J. Lally

**Affiliations:** 1 Graduate Entry Medicine, Royal College of Surgeons in Ireland, Dublin, Ireland; 2 Director of Pharmacy and Pathology, South London and Maudsley NHS Foundation Trust, London, UK; 3 King’s College London, Institute of Psychiatry, Psychology and Neuroscience, London, UK; 4 National Psychosis Service, South London and Maudsley NHS Foundation Trust, London, UK; 5 Department of Psychosis Studies, Institute of Psychiatry, Psychology and Neuroscience, London, UK; 6 Department of Psychiatry, Royal College of Surgeons in Ireland, Dublin, Ireland; 7 St Vincent’s Hospital Fairview, Dublin, Ireland; 8 Department of Psychiatry, Mater Misericordiae University Hospital, Dublin, Ireland

**Keywords:** 25 (OH)D, antipsychotics, immune, pneumonia, psychosis

## Abstract

Vitamin D deficiency is associated with an increased risk of acute respiratory infection. There is an excess of respiratory infections and deaths in schizophrenia, a condition where vitamin D deficiency is especially prevalent. This potentially offers a modifiable risk factor to reduce the risk for and the severity of respiratory infection in people with schizophrenia, although there is as yet no evidence regarding the risk of COVID-19. In this narrative review, we describe the prevalence of vitamin D deficiency in schizophrenia, report the research examining the relationship between vitamin D levels and COVID-19 and discuss the associations between vitamin D deficiency and respiratory infection, including its immunomodulatory mechanism of action.

## Introduction

In December 2019, a novel coronavirus, severe acute respiratory syndrome coronavirus 2 (SARS-CoV-2), emerged in Wuhan, Hubei province, China. The World Health Organization (WHO) declared coronavirus disease 2019 (COVID-19) a pandemic on 11 March 2020. While it is estimated that 80% of those with COVID-19 are asymptomatic or have a self-limiting disease, the case fatality rate for those hospitalised with COVID-19 is 2.3%, increasing to 10.5% in those with cardiovascular disease, 7.3% in those with diabetes mellitus and 6% in those with hypertension (Wu & McGoogan, 2020).

People with schizophrenia have an excess of physical co-morbidities and reduced life expectancy (Hjorthøj *et al.* 2017), and may be an especially vulnerable group to COVID-19 disease and increased mortality (Kozloff *et al.* 2020). Patients with schizophrenia have a higher burden of the risk factors identified above (Gardner-Sood *et al.* 2015; Gaughran *et al.* 2019; Lally, Spaducci, *et al.* 2019), leaving them more vulnerable to adverse outcomes of infection. There is also an excess of deaths from pneumonia and influenza in people with schizophrenia (Olfson *et al.* 2015a). Exploring possible protective factors to mitigate risk, therefore, is essential for this vulnerable patient group.

Vitamin D is known to have a critical role in the immune system, influencing many functions of the normal immune response to pathogens. In those who are vitamin D deficient, immune function is disrupted in favour of a pro-inflammatory state. Dysregulated inflammation and ‘cytokine storms’ are associated with worse outcomes in COVID-19, contributing to the severity of acute respiratory distress syndrome (ARDS) and organ dysfunction (Cao, 2020). Early data currently available only as preprints suggest a major effect for vitamin D status on COVID-19 outcome (Alipio, 2020; Raharursan, 2020). The prevalence of vitamin D deficiency in schizophrenia is high, therefore, one potential stratagem to mitigate the risk of COVID-19 in schizophrenia may be to optimise vitamin D concentrations. This study reviews the association between vitamin D deficiency and the risk of infection and provides suggestions for optimising vitamin D in people with schizophrenia.

## Background

The two main forms of vitamin D are vitamin D3, or cholecalciferol, which is formed in the skin after exposure to sunlight (ultraviolet B (UVB) radiation) and ergocalciferol, or vitamin D2, which is obtained by UV irradiation of plants or plant sources. Vitamin D has a central role in bone mineralisation and its primary function is to aid the intestinal absorption of calcium and phosphate (Holick, 2012).

There is long-standing evidence linking vitamin D deficiency with skeletal disease, including rickets (Holick, 2006), osteomalacia, muscle weakness (Redzic*et al*. 2013), falls, osteoporosis and fractures (Redzic *et al*. 2013; Bolland *et al*. 2018) (Table 1). Increasing observational and epidemiological data have identified associations between vitamin D deficiency and non-skeletal diseases, including infectious diseases (Bouillon *et al.* 2019).

**Table 1.** Classification of vitamin D status for skeletal health

For the ranges below, vitamin D status is measured in terms of 25(OH)D serum concentration
Most common thresholds^a^
Deficiency: <50 nmol/l (20 ng/ml)
Insufficiency: 51–74 nmol/l (21–29 ng/ml)
Sufficiency: >75 nmol/l (>30 ng/ml)
Most conservative thresholds^b^
Deficiency: <25 nmol/l (<10 ng/ml)
Insufficiency: 25–50 nmol/l (10–20 ng/ml)
Sufficiency: >50 nmol/l (>20 ng/ml)

a Holick & Chen (2008); US Institute of Medicine: Ross *et al.* (2011).

b International Osteoporosis Foundation: Dawson-Hughes et al. (2010).

Ecological evidence is suggestive of links between seasonal vitamin D deficiency and increased respiratory infection risk (Grant, 2008). Further, preliminary epidemiological data points to increased mortality rates from COVID-19 in populations living above latitude 35° North, compared to those in countries below 35° North, this coincides with those countries in which vitamin D deficiency is more prevalent due to inadequate sunlight exposure in Winter months (Rhodes *et al.* 2020). In this narrative review, we will describe the prevalence of vitamin D deficiency in the first episode and established psychosis, and the associations between suboptimal vitamin D and respiratory tract infections. We will provide an analysis of the potential benefits of vitamin D supplementation as a preventative measure for those with schizophrenia at risk of vitamin D deficiency associated respiratory infection and COVID-19.

## Prevalence of vitamin D deficiency in schizophrenia

People with schizophrenia and other psychotic disorders are at high risk for vitamin D deficiency. A cross-sectional study of community-based patients in the UK identified that 90% of people with established psychosis had suboptimal levels of vitamin D (<20 ng/ml (<50 nmol/l)), with a vitamin D deficiency (<10 ng/ml (<25 nmol/l)) rate of 50%, much higher than the rate of 15% rate in the UK general population (Ruston *et al.* 2004). Rates of vitamin D deficiency of 49% were identified in the UK hospital population of patients with schizophrenia (Patel & Minajagi 2018). Comparable vitamin D deficiency rates have been identified in the first-episode psychosis (Lally, Ajnakina, *et al.* 2019), with the risk of vitamin D deficiency in individuals with FEP being threefold higher than in their age, gender and ethnicity-matched controls (Crews *et al.* 2013). Consequences of low vitamin D, including the relationship with infection risk in this population are unclear as of yet and require further research.

## Vitamin D and respiratory infection risk

There is an inverse relationship between serum vitamin D concentrations and the risk of acute respiratory tract infections (Martineau *et al.* 2017). A recent meta-analysis of eight observational studies (*n* = 21 000) identified that individuals with a vitamin D level <20 ng/ml (i.e. <50 nmol/l) had a 64% increased risk of community-acquired pneumonia (Zhou *et al.* 2019).

There is evidence that vitamin D supplementation improves immune function and reduces the risk of respiratory infection, with higher vitamin D levels reducing the severity of influenza and respiratory infections(Grant & Giovannucci, 2009; Martineau *et al.* 2017, 2019; Gruber-Bzura, 2018; Huang *et al.* 2020). A meta-analysis of individual participant data from 25 randomised controlled trials, reporting on 10 933 participants, found that vitamin D supplementation was well tolerated and provided modest protection against acute respiratory tract infections (adjusted OR 0.88; 95% confidence interval (CI) 0.81–0.96) (Martineau *et al.* 2017). The effects of vitamin D supplementation are more significant in those with vitamin D deficiency, with vitamin D supplementation reducing the risk of acute respiratory infection (Martineau *et al.* 2017). This is relevant to people with schizophrenia, the majority of whom have suboptimal vitamin D concentrations. This raises the possibility that vitamin D supplementation may not only provide skeletal benefits, but may contribute to protection against and reduction in the severity of respiratory infections, which may extend to SARS-CoV2.

## Mechanism

Observational studies have identified associations between suboptimal vitamin D levels and increased respiratory infection risk, including SARs-CoV2 infection (Alipio, 2020; Lau *et al.* 2020), but do not attribute causation. There is, however, pre-clinical data providing mechanistic evidence of how vitamin D impacts on risk for viral infections (Grant *et al.* 2020).

### Vitamin D function in the normal immune response

The availably of adequate serum concentration of 25(OH)D is important in mounting an immune response and enhancing immunomodulation, while the absence of adequate vitamin D may lead to an aberrant response to pathogens or autoimmunity (Lang *et al.* 2013). The majority of immune cells express vitamin D receptor (VDR), and vitamin D has a role in immunomodulation, influencing antigen presentation, innate immunity and T-cell function (Lang & Aspinall, 2017). Vitamin D also affects the expression of angiotensin-converting enzyme 2 (ACE2), the functional receptor of which has been identified as the entry site for the SARS-CoV-2 (Lu *et al.* 2020; Walls *et al.* 2020).

Activated vitamin D (1,25-dihydroxyvitamin D (1,25(OH)_2_D)) has a role in both innate and adaptive immunity. Macrophages and dendritic cells express VDR, and their function is responsive to circulating activated vitamin D (Chun *et al.* 2014). In response to a pathogen challenge, macrophages express increased VDR and 1*α*-hydroxylase (CYP27B1), which then converts biologically inert vitamin D into its active form. The binding of VDR also inhibits the production of pro-inflammatory cytokines (Lang & Aspinall, 2017). Vitamin D has been shown to inhibit the development of pro-inflammatory Th17 cells and initiate the expression of regulatory T cells that suppress inflammation and defend against autoimmunity (Lang *et al.* 2013; Ghavideldarestani *et al*. 2020).

### Vitamin D regulating inflammation

Vitamin D is proposed to have anti-inflammatory effects. Vitamin D supplementation is associated with a reduction in C-reactive protein (CRP) in the general population (Chen *et al.* 2014), while in schizophrenia, an inverse relationship between vitamin D and CRP levels was identified (Lally *et al.* 2016). An unblinded RCT of vitamin D supplementation with 50 000 IU vitamin D3 (combined with probiotic supplements) in people with chronic schizophrenia demonstrated reductions in CRP and enhanced total antioxidant capacity compared to placebo (Ghaderi *et al.* 2019). Thus, vitamin D’s suggested anti-inflammatory effects may have a protective role against the severity of acute respiratory infection in COVID-19.

An animal model study identified the beneficial effects of calcitriol, the biologically active form of vitamin D, in lipopolysccacaride (LPS)-induced lung injury. LPS is a glycoprotein with potent pro-inflammatory effects, inducing a strong inflammatory state and specifically damaging pulmonary vascular endothelial cells, resulting in acute lung injury and ARDS (Xu *et al.* 2017). The mechanism by which calcitriol produced these beneficial effects was via manipulation of the renin–angiotensin system (RAS), balancing the opposing effects of ACE and ACE2 in favour of low endovascular permeability.

Optimising serum 25 (OH)D concentration is associated with responsive downregulation via induction of regulatory T cells and regulation of cathelicidin-encoding gene expression (Lang & Aspinall, 2017; Cao, 2020; Mccartney & Byrne, 2020). Cathelicidins are stimulated by infection to produce powerful cytokines including IL-1, IL-6 and INF-gamma, vitamin D has a role in regulating this pro-inflammatory process (Lang & Aspinall, 2017). The immunomodulatory effect of vitamin D could, therefore, attenuate some of the downstream inflammatory sequelae associated with poor clinical outcomes in severe respiratory illnesses including COVID-19 infection. These include persistent IL-6 elevation and prolonged interferon-gamma (INFγ) response as well as high levels of other pro-inflammatory cytokines, a state termed “cytokine storm” (Cao, 2020).

### Vitamin D and COVID-19 host cell invasion

The ACE 2 receptor has been identified as the primary site for host cell invasion for COVID-19. ACE 2 is expressed in the airway epithelia and lung parenchyma, as well as digestive system organs. ACE 2 converts angiotensin II (Ang II) into angiotensin 1–7, which acts as a vasodilator with a regulatory role in the RAS. Vitamin D may suppress RAS activity via inhibition of renin and the ACE/Ang II/AT1R cascade (Ghavideldarestani *et al*. 2020). In the absence of adequate 25 (OH)D serum concentration, this function can become less efficient, potentially contributing to effects such as increased inflammation and thrombotic events (Xu *et al.* 2017).

In Middle Eastern Respiratory Syndrome (MERS) 2, a molecular virulence mechanism including dipeptidyl peptidase-4 receptor (DPP-4/CD26) binding was identified. While the DPP-4/CD26 receptor has not been confirmed as a target for COVID-19, the genetic similarity of MERS 2 and COVID-19 (Walls *et al.* 2020), justifies considering this receptor a putative secondary adhesion molecule for COVID-19 host cell invasion (Mccartney & Byrne, 2020). In pre-clinical models, correction of vitamin D deficiency is associated with reduced DPP-4/CD26 receptor expression(Komolmit *et al.* 2017).

### Summary

**Fig. 1.**
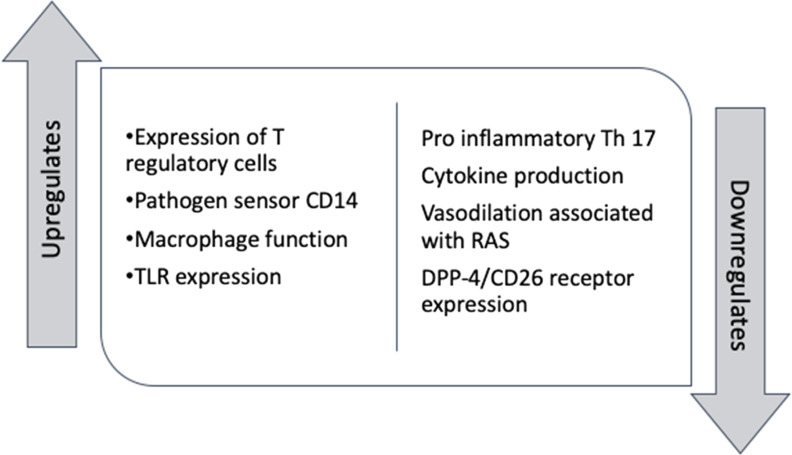
Mechanism of vitamin D anti-inflammatory and immunomodulatory function.

The mechanisms by which vitamin D exerts its role in the immune system are multifactorial (Fig. 1). Low serum vitamin D concentration seems to be associated with inhibition of normal immunomodulatory function in favour of pro-inflammatory state. Inadequate vitamin D levels may result in less efficient macrophage function and antigen presentation. Low vitamin D may, therefore, potentially contribute to a delayed or dysregulated response to the body’s initial contact with SARS-CoV-2 or impede the mounting of an appropriate defence in established SARS-CoV-2 infection.

## Vitamin D optimisation strategies

Vitamin D is mainly acquired from sunlight exposure, with a smaller amount gained from dietary sources (approximately 10%). Although there is variation in the definition of vitamin D deficiency (Table 1), in Ireland, approximately 50% of both older (>55 years) and adult age populations have vitamin D insufficiency (<50 nmol/l) (Laird & Anne Kenny 2020) and people with schizophrenia are a high-risk group for vitamin D deficiency (see Table 2).

**Table 2.** Risk factors for developing vitamin D deficiency

Biological/environmental
Pigmented skin
Increased age
Inadequate sunlight exposure
Clothing concealing skin
Sunscreen with sunscreen protection factor >30
Dietary insufficiency in context of inadequate sunlight exposure
Vegan (e.g. no fish in diet)
Long-term hospitalisation/institutionalisation
Medical
Obesity
Pregnancy:
Multiple short interval pregnancy
Fat malabsorption syndrome (Coeliac disease; Crohn’s disease; cystic fibrosis; liver disease)
Short bowel syndrome
Bariatric surgery
Schizophrenia and other psychiatric disorders
Primary hyperparathyroidism
Chronic granulomatous disorders: Sarcoidosis
Tuberculosis
Chronic fungal infections
Liver disease
Nephrotic syndrome
Medication
Anticonvulsants:
Carbamazepine
Phenytoin
Rifampicin
Cholestyramine
Glucocorticoids
Highly active antiretroviral treatment (HAART)

The current recommendation for the prevention of vitamin D deficiency in adults in Ireland is daily vitamin D3 supplementation of 5µg (200 IU) and 10–15 µg (400–600 IU/day) for the high-risk groups according to healthy eating guidelines set out by the Food Safety Authority of Ireland (Food Safety Authority of Ireland, 2011). There is a differential dose response to vitamin D, with lower doses such as 400 –600 IU/day more suited to prevent vitamin D deficiency, while higher doses are required to treat vitamin D deficiency.

Significant variations have been noted in the dose–response relationship when attempting to supplement to achieve sufficient serum vitamin D concentrations (Aloia *et al.* 2008). The dose–response data indicates the tolerability and safety of high-dose vitamin D3 supplementation, with more rapid correction of vitamin D concentrations and increased likelihood of attaining optimal vitamin D concentrations seen with high doses. Daily vitamin D intake of up to 10 000 IU per day is felt to be safe, and recommendations for up to 4000 IU per day in adults have been made (Holick *et al.* 2011; Ross *et al.* 2011). In the absence of cutaneous sunlight exposure, adult males are shown to require 4000 IU per day to maintain vitamin D concentrations (Heaney *et al.* 2003). In a general population cohort study ,vitamin D3 at 4000 IU/day was effective in raising serum 25(OH)D to >40 ng/ml in all adults over a duration of up to 5 months, with no safety concerns (range of vitamin D values post-supplementation 69–125 nmol/l) (Vieth *et al*. 2001).

There is little data on vitamin D supplementation and dose response in schizophrenia, meaning that recommendations are based on general population data. It is pertinent that some people with schizophrenia may be unable to implement strategies to ensure adequate vitamin D levels through sunlight exposure and outdoor activity (e.g. those who are hospitalised).

In one of the few reports in mental health settings, long-term high-dose vitamin D supplementation was prescribed for the majority of admissions to a psychiatric hospital in Cincinnati, Ohio with no adverse events reported (McCullough *et al*. 2019). All newly admitted patients were offered 5000–10 000 IU vitamin D3 per day. Analysis of data on 36 inpatients who received 5000 IU/day for 12 months or longer showed an increase in the mean serum 25(OH)D concentration from 24 to 68 ng/ml (60–170 nmol/l), whereas for the 78 patients who received 10 000 IU/day, mean concentrations increased from 25 to 96 ng/ml (62.5–240 nmol/l) (McCullough *et al*. 2019).

Evidence from general population studies, therefore, indicates that higher doses of vitamin D supplementation may be needed (e.g. 4000 IU/day) to treat deficiency, that such high doses are well tolerated and that the individual response may be dependent on the baseline 25(OH)D concentrations (Ross *et al.* 2011).

## Potential benefits of vitamin D supplementation in schizophrenia during the COVID-19 pandemic

People with schizophrenia have an increased prevalence of pneumonia and respiratory tract infections (Dzahini *et al.* 2018) with associated excess mortality, with a standardised mortality ratio (SMR) from influenza and pneumonia of 7.0 compared to the general population (Olfson *et al.* 2015b). Observational data shows that low serum vitamin D levels, and particularly vitamin D deficiency, are associated with a higher incidence of acute respiratory tract infections (Pham *et al.* 2019) and an increase in respiratory symptoms in adult populations (Mulrennan *et al.* 2018; Hong *et al.* 2020)

To date, there is no evidence of a specific effect of vitamin D for the treatment or prevention of COVID-19 (Lee, 2020). The recommendation remains that clinicians should treat vitamin D deficiency irrespective of any link with a respiratory infection (Lee, 2020).

However, data relating to this question is emerging at pace. Preliminary reports have shown inverse correlations between serum vitamin D concentrations and more severe respiratory infections and mortality due to SARS-Cov2, with populations that are deficient (<30 nmol/l) being most at risk (Alipio, 2020; Ilie *et al*. 2020; Lau *et al.* 2020; Raharursan, 2020). On the other hand, an early analysis of the UK Biobank data found no association between vitamin D levels taken in 2006–2010 and subsequent infection with COVID-19 (Hastie *et al.* 2020).

Significant effects of vitamin D supplementation in reducing the risk of non-COVID-19 respiratory tract infections for those with lower vitamin D levels were identified in a systematic review of 7 meta-analyses of 30 RCTs (Rejnmark *et al.* 2017). A meta-analysis of 11 RCTs of 5389 patients found that vitamin D supplementation was associated with a 40% reduction in the risk for ARTIs (Bergman *et al.* 2013). Subgroup analysis found that daily administration of vitamin D3 was significantly associated with a reduction in ARTIs compared to less frequent administration, and with no effect seen for once-monthly bolus administration (Bergman *et al.* 2013). An RCT in 124 subjects found a significantly increased probability of remaining free from respiratory tract infections and fewer respiratory tract infections in those subjects treated with 4000 IU vitamin D3 per day compared to a placebo-treated group (Bergman *et al.* 2015).

## Vitamin D supplementation in Schizophrenia

There is a paucity of data on vitamin D supplementation in schizophrenia, with two RCTs investigating the effects of vitamin D3 supplementation on psychotic symptoms, cognition and cardiometabolic outcomes (Krivoy *et al.* 2017; Ghaderi *et al.* 2019), and one further RCT underway (Gaughran *et al.* 2020), but none investigating the effects in respiratory infections.

People with schizophrenia are more likely to have vitamin D deficiency or insufficiency (Lally *et al.* 2016; Adamson *et al.* 2017; Zhu *et al.* 2020). A small-cross sectional study in clozapine-treated patients identified inverse correlations between vitamin D concentrations and the pro-inflammatory interleukin-6 (IL-6), possibly indicative of the immunomodulatory effect of vitamin D in this population (Krivoy *et al.* 2020). However, there is insufficient evidence to stratify vitamin D supplementation, given that suboptimal vitamin D concentration is the norm in this population.

When considering vitamin D testing an initial presumptive diagnosis of insufficiency could be made, as is the case for high-risk groups in the general population, based on risk factors, without the need for (expensive) testing of vitamin D levels unless symptomatic (Lally & Gaughran, 2019; Burton *et al.* 2010). Indeed, some patients with active psychosis may decline testing and need to be treated presumptively, taking any safety precautions into account. In the longer term, the ideal approach to vitamin D supplementation in people with schizophrenia may be ensuring prophylactic administration in line with national guidelines. However, standard recommendations for vitamin D supplementation may be inadequate to normalise vitamin D levels in this group, who may be unable to implement lifestyle changes to enhance outdoor activity and vitamin D synthesis. It may, therefore, be important to take levels to assess the effect of supplementation.

Furthermore, current vitamin D treatment recommendations are to maintain vitamin D concentrations for the purpose of maintaining skeletal health. It is unclear, however, what the optimal levels are for protection against respiratory and viral infections. Observational data indicates that 70–90% of those with schizophrenia have vitamin D concentrations below the threshold (50 nmol/l) (Lally *et al.* 2016; Zhu *et al.* 2020) at which respiratory tract infection risk is known to increase (Pham *et al.* 2019).

Lifestyle advice should be offered to all patients, and education that the best source for vitamin D is sensible levels of sunlight exposure. Spending 10–15 minutes in the sunlight at least twice a week, with face and arms exposed, will suffice to ensure adequate vitamin D levels(Nowson *et al.* 2012), bearing in mind however that some psychotropic medications are associated with photosensitivity.

In treating vitamin D deficiency, the frequency of administration seems to be relevant. A meta-analysis of individual patient data identified that the protective effect of vitamin D in respiratory tract infections was significantly greater in those receiving daily or weekly doses compared to larger, less frequent bolus doses, with the effects strongest in those with vitamin D deficiency (<25 nmol/l) who received daily or weekly doses (Martineau *et al.* 2017).

In patients with schizophrenia, most studies have shown suboptimal vitamin D levels, including in community patients. For these patients, in the absence of contraindications (see below), 4000 IU daily of vitamin D3 (with a lower dose of 2000 IU in the elderly) may be needed for a period of at least 4 weeks and up to a maximum of 12 weeks. A maintenance regimen could then be initiated guided by levels. A 4-week period of 4000 IU daily vitamin D supplementation may suffice in the Spring and Summer months in the European countries, as there remains a potential for cutaneous vitamin D synthesis in the Summer months. A longer period of supplementation may be more appropriate in the Autumn and Winter months (up to 12 weeks). These recommendations may be more relevant to inpatients and those in long-term care residential facilities, settings in which increased morbidity and mortality secondary to the level of COVID-19 have been identified.

In adults with vitamin D deficiency, vitamin D3 supplementation will not lead to a normalising of 25 (OH)D concentrations until an 8–12-week period of adequate dosing is complete, so vitamin D levels should not be checked for at least 12 weeks after treatment initiation. It is appropriate to check baseline serum calcium and at 4 weeks to assess for hypercalcaemia and an unmasked primary hyperparathyroidism.

Caution or referral for specialist opinion is recommended if there is evidence or a history of hypercalcaemia, a history of granulomatous disorders (Sarcoidosis, tuberculosis), a history of lymphoma, a history of renal calculi or renal impairment with eGFR < 60 ml/min. Nephrology advice is recommended for those with renal impairment, as 1,25-dihydroxyvitamin D may be required.

## Vitamin D toxicity

While rare, vitamin D toxicity can cause severe hypercalcaemia when ingested at excessive amounts over a long period of time (Heaney *et al.* 2003). Serum 25(OH)D concentrations would need to exceed 150 ng/ml (375 nmol/l) before toxicity may become an issue, and the risk of hypercalcaemia is mitigated by ensuring vitamin D levels remain below 100 ng/ml (250 nmol/l) (Holick *et al.* 2011). Cohort studies have shown that dosing up to 40 000 IU per day over 28 weeks did not cause an increase in adverse effects, and supplementation at 5500 IU for 20 weeks did not raise serum concentrations above 160 nmol/l (Holick *et al.* 2011). The risk for vitamin D-induced hypercalcaemia is low, with rates of transient mild hypercalcaemia (2.56–2.64 mmol/l) of 3% and no severe hypercalcaemia in a population of healthy adults aged 55–70 treated with 4000 IU/day for 3 years (Billington *et al.* 2019).

## Conclusion

With the advent of a new respiratory pandemic, SARS-Cov2, that as of yet does not show consistent response to antiviral therapy and with no vaccine immediately available, it is important to optimise the underlying health state of patients with schizophrenia. The importance of this question in people with schizophrenia is amplified by their increased likelihood of exposure to infection in hospital settings and the high rates of COVID-19-relevant co-morbidities. Further evidence is urgently needed regarding the effectiveness of vitamin D optimisation as a protective measure in this group.

This review presents the evidence that optimisation of vitamin D could be beneficial to those with schizophrenia and other psychotic disorders through possible protective effects against respiratory tract infections. While specific optimisation strategies have not been devised for those with schizophrenia, it is reasonable to adapt the general population guidelines, bearing in mind the greatly enhanced likelihood of vitamin D deficiency in those with psychotic disorders.

The prophylactic use of vitamin D supplementation to at-risk groups such as patients with schizophrenia may prove to be a cost-effective measure to improve resilience to respiratory infections. It is possible that this effect may extend to SARS-Cov2 infection, although clinical trial evidence supporting this is not yet available.
